# “Don’t judge me!”: Links between in vivo attention bias toward a potentially critical judge and fronto-amygdala functional connectivity during rejection in adolescent girls

**DOI:** 10.1016/j.dcn.2021.100960

**Published:** 2021-05-05

**Authors:** Stefanie L. Sequeira, Dana K. Rosen, Jennifer S. Silk, Emily Hutchinson, Kristy Benoit Allen, Neil P. Jones, Rebecca B. Price, Cecile D. Ladouceur

**Affiliations:** a University of Pittsburgh, Department of Psychology, United States; b University of Tennessee, Department of Psychology, United States; c University of Pittsburgh, Department of Psychiatry, United States

**Keywords:** Adolescence, Attention bias, Functional connectivity, Social rejection

## Abstract

During adolescence, increases in social sensitivity, such as heightened attentional processing of social feedback, may be supported by developmental changes in neural circuitry involved in emotion regulation and cognitive control, including fronto-amygdala circuitry. Less negative fronto-amygdala circuitry during social threat processing may contribute to heightened attention to social threat in the environment. However, “real-world” implications of altered fronto-amygdala circuitry remain largely unknown. In this study, we used multiple novel methods, including an in vivo attention bias task implemented using mobile eye-tracking glasses and socially interactive fMRI task, to examine how functional connectivity between the amygdala and prefrontal cortex (PFC) during rejection and acceptance feedback from peers is associated with heightened attention towards potentially critical social evaluation in a real-world environment. Participants were 77 early adolescent girls (ages 11–13) oversampled for shy/fearful temperament. Results support the reliability of this in vivo attention task. Further, girls with more positive functional connectivity between the right amygdala and anterior PFC during both rejection and acceptance feedback attended more to potentially critical social evaluation during the attention task. Findings could suggest that dysfunction in prefrontal regulation of the amygdala’s response to salient social feedback supports heightened sensitivity to socially evaluative threat during adolescence.

## Highlights

• We used innovative, ecologically valid eye-tracking and fMRI measures to examine social threat sensitivity in adolescent girls.
• Findings support the reliability of a novel in vivo attention bias task.
• Real-world attentional biases toward social threat correlated with amygdala-anterior PFC functional connectivity during social evaluation.
• Greater positive amygdala-anterior PFC connectivity during social evaluation could suggest disrupted prefrontal regulation of the amygdala.
• Disrupted prefrontal regulation of the amygdala could contribute to deployment of attention to social evaluative threat in daily life.

## 1 Introduction

Early adolescence is marked by significant increases in social sensitivity, or increased attentional and emotional processing of information regarding social evaluation (Somerville, 2013). Heightened social sensitivity is crucial for adolescents to navigate and learn from changing social environments. The ability to distinguish which attitudes and behaviors are accepted versus rejected by peers is key for helping adolescents “fit in;” this ability not only helps adolescents choose peers to align with, but also to develop their individual identities within peer groups.

While social sensitivity is a normative part of adolescent development, heightened sensitivity to negative social evaluation may contribute to elevated rates of social anxiety in adolescence (Silk et al., 2012a). Moreover, preferential attention towards social threat (both hypervigilance and difficulty disengaging attention from threat) could be one component of heightened sensitivity to negative social evaluation that contributes to the onset and maintenance of anxiety (Van Bockstaele et al., 2014). Heightened sensitivity to negative social evaluation in adolescence may be supported by perturbations in amygdala-prefrontal cortex (PFC) circuitry, given the role of this circuitry in detecting and regulating negative emotions, as well as its role in attentional processes (Cisler and Koster, 2010; Pine and Fox, 2015; LeDoux and Pine, 2016). However, the extent to which reduced amygdala-PFC coupling contributes to adolescents’ responses to potential negative evaluation in the real world remains unknown. The present study thus aimed to examine the association between amygdala-PFC functional connectivity during peer social evaluation and attention biases during social evaluation in vivo. We focused this study on adolescent girls oversampled for shy/fearful temperament, a population with high sensitivity to social evaluation (Rudolph and Conley, 2005) and heightened risk for social anxiety disorder (Merikangas et al., 2010; Chronis-Tuscano et al., 2009). Greater insight into the biobehavioral correlates of sensitivity to negative social evaluation in this sample may elucidate antecedents of social anxiety during a key developmental window and inform targets for future intervention.

Heightened sensitivity to negative social evaluation in adolescence may be supported by developmental changes in fronto-amygdala circuitry. The amygdala plays a key role in detecting emotional information in the environment, including socially threatening information, and helps facilitate attention to salient stimuli through connections with sensory pathways (Phelps and LeDoux, 2005). The amygdala also has dense structural connections with ventromedial portions of the PFC (Ghashghaei et al., 2007; Ray and Zald, 2012), many of which inhibit amygdala activity (Etkin et al., 2011). More lateral regions of the PFC may also interact with the amygdala to support cognitive processes, such as attention regulation (Pine and Fox, 2015; LeDoux and Pine, 2016). The amygdala and PFC each change in structure and function during adolescence (Spear, 2000; Hare et al., 2008; Schumann et al., 2004). Multiple early models of adolescent brain development suggest that the amygdala (and other subcortical structures) develops early, followed by protracted development of the prefrontal cortex (e.g., Ernst et al., 2006; Nelson et al., 2005; Casey et al., 2008).

Building on these early models is more recent research examining the development of fronto-amygdala functional connectivity. At a circuit level, multiple studies have shown that functional connectivity between the amygdala and medial PFC during threat processing is more negative in adolescents and adults relative to children (Gee et al., 2013; Wu et al., 2016; Silvers et al., 2017). Negative fronto-amygdala coupling during threat processing could represent greater “top-down” prefrontal control over a “bottom-up” amygdala fear response, which could support successful emotion regulation (Banks et al., 2007; Phillips et al., 2008; Robinson et al., 2019), both at the implicit/automatic level (e.g., Gee et al., 2013) and at the explicit/conscious level (e.g., Silvers et al., 2017). In support of this interpretation, research in adolescents has shown associations between more negative amygdala-PFC connectivity during threat and better cognitive control and lower negative emotionality (Davis et al., 2019), as well as use of less avoidance strategies in response to negative interactions in daily life (Price et al., 2016).

On the other hand, less negative fronto-amygdala connectivity during threat processing may place children and adolescents at risk for anxiety. A substantive literature has linked youth anxiety to altered fronto-amygdala connectivity while viewing negative emotional stimuli (e.g., Monk et al., 2008; Strawn et al., 2012; Campbell-Sills et al., 2011; White et al., 2017; Abend et al., 2020). Much of this research suggests that youth with anxiety show less negative coupling (or more positive coupling) between the amygdala (particularly the right amygdala) and regions of ventrolateral and medial PFC in response to social threat (Monk et al., 2008; Davis et al., 2019) and non-social threat (Strawn et al., 2012). However, some studies have found associations between anxiety risk or anxiety symptoms in youth and greater negative connectivity between the amygdala and dorsolateral PFC when processing threats (e.g., Hardee et al., 2013; Abend et al., 2020). Many of these studies use the dot-probe task (MacLeod et al., 1986), a widely-used computer paradigm that uses stationary stimuli to assess attention biases towards threatening stimuli, such as fearful faces, relative to more neutral stimuli. Less negative fronto-amygdala coupling on the dot-probe task could reflect either hypervigilance towards threat or difficulty disengaging attention from threat, which may confer risk for anxiety.

Taken together, findings suggest that more negative coupling between the amygdala and PFC during threat processing in adolescence may support better emotion regulation, including better attentional control in the context of emotional stimuli (Gross, 2014; Bishop, 2008). Less negative amygdala-PFC connectivity might confer risk for anxiety in part due to poorer attentional control in the presence of threat, which contributes to preferential attention towards threats (Cisler and Koster, 2010). Supporting this hypothesis is a growing body of behavioral research showing that youth with anxiety and at temperamental risk for anxiety have attentional biases towards threat, typically assessed behaviorally using reaction time measurements, or more recently, using eye-tracking indices (Rosen et al., 2019; Pérez-Edgar et al., 2010; Dudeney et al., 2015; Van Bockstaele et al., 2014).

A major limitation of the attention bias literature, however, is that the reliability, ecological validity, and generalizability of current attention bias tasks, especially the computerized dot-probe task, have been called into question (MacLeod, Grafton, & Notebaert, 2019; Price et al., 2015). There is increasing concern that the growing body of research that uses dot-probe tasks may lack reliability given the low internal consistency and test-retest reliability that fail to meet psychometric standards, particularly when examining individual differences (MacLeod et al., 2019; Price et al., 2015). Furthermore, attentional patterns have been shown to be different in computer-based tasks compared to socially-interactive, real-world settings (Kretch and Adolph, 2015). Unlike real-world settings, computer-based tasks employ static images presented for brief intervals and attempt to experimentally direct attention (Allen et al., 2019). These concerns regarding computer-based attention tasks point to a need for more ecologically valid and reliable paradigms. Importantly, these are now becoming available given recent technological advances in eye-tracking, which have moved beyond computer-based paradigms and enable the measurement of attention biases during in vivo social interaction (e.g., Allen et al., 2019; Hutchinson et al., 2019; Woody et al., 2019). Mobile eye-tracking paradigms have the potential to elucidate the nature and time course of attention biases within a real-world social environment, thereby enhancing ecological validity compared to dot-probe paradigms.

The present study capitalizes on these technological advances to examine real-world attentional patterns. We used a novel in vivo interpersonal stress task to study attention towards potentially critical social feedback versus positive social feedback while adolescent girls gave a speech wearing mobile eye-tracking glasses. We focused our study on girls given evidence of heightened social sensitivity (Guyer et al., 2009) and emotional reactivity to social stress (Rudolph et al., 2016) in this population. Addressing critical limitations in the literature, we examined the reliability of this task. Assuming adequate reliability, attention bias scores (i.e., difference between attention to critical/negative feedback relative to positive feedback) were then tested as predictors of fronto-amygdala functional connectivity during a separate social rejection fMRI task. This allowed for testing the primary research question: How do girls with less negative amygdala-PFC functional connectivity to social threat engage with social threat in the real world? We hypothesized that less negative, or more positive, amygdala-PFC coupling during social rejection would be associated with preferential attention to negative social evaluation in vivo in adolescent girls at risk for anxiety. Findings from this research could provide insight into mechanisms supporting heightened sensitivity to potential social evaluative threat in adolescence. To test specificity, we also examined associations between real-world attentional biases and fronto-amygdala functional connectivity during social acceptance.

## 2 Methods

### 2.1 Participants

One-hundred-twenty-nine early adolescent girls ages 11–13 were recruited for participation in the study via online advertisements and announcements in the community. Girls were recruited based on parent-reported sex at birth; gender identity was not assessed at recruitment. This study oversampled for shy/fearful temperament, a risk factor for the development of social anxiety in adolescence and adulthood (Chronis-Tuscano et al., 2009), to enrich variability in threat responsivity. Temperament was assessed prior to participants’ first visit using the Early Adolescent Temperament Questionnaire- Revised (EATQ-R; Ellis and Rothbart, 2001). The EATQ-R was designed to measure temperament traits in early adolescence (ages 9–15), with items specific to adolescent life experiences. To determine temperament status, participants were compared against established distribution scores of the EATQ-R shyness and fear scales (Ellis and Rothbart, 2001). The sample was stratified such that approximately 2/3 of participants (*n* = 85) scored > 0.75 SDs above the mean on the parent- or adolescent-rated fear scales (3.12 for parent-report, 3.48 for adolescent-report) or shyness scales (2.99 for parent-report, 3.16 for adolescent-report). All other participants (*n* = 44) scored below this cut-off and were considered to be in the normative range of shy/fearful temperament.

To be eligible for the study, participants could not meet DSM-5 criteria for a current or lifetime diagnosis of any anxiety disorder (except for specific phobia), obsessive-compulsive disorder, post-traumatic stress disorder, major depressive disorder, or any psychotic or autism spectrum disorder, as determined by the Kiddie-Schedule for Affective Disorders and Schizophrenia (K-SADS-PL; Kaufman, Birmaher, Axelson, Perepletchikova, Brent & Ryan, 2016). In addition, participants had an IQ > 70, as assessed using the Wechsler Abbreviated Scale of Intelligence (WASI; Wechsler, 1999). Additional exclusionary criteria include a lifetime presence of a neurological or serious medical condition, the presence of any MRI contraindications, presence of head injury or congenital neurological anomalies (based on parent report), acute suicidality, taking medications that affect the central nervous system (e.g., selective serotonin reuptake inhibitors), and ocular conditions that would impede eye tracking measurement and/or ability to see clearly without prescription glasses. Stimulants were permitted if use was discontinued for 36 h prior to the scan.

Two participants (one high in shy/fearful temperament and one in the normative range) dropped out of the study before the eye-tracking tasks because of time commitments, leaving a sample of 127 participants who attempted the Attention Speech Task (AST). Of these participants, 108 had usable data. Participants were excluded from the AST analysis because they did not complete the task due to distress about public speaking (*n* = 3), data were lost due to disconnection with the Tobii program (*n=*6), were from a sibling pair (*n* = 1), had less than 50 % valid gaze data (i.e., where gaze coordinates could be estimated by Tobii) (*n* = 7), had an ocular condition (*n* = 1), or did not have sufficient visual acuity without glasses (*n* = 1). Participants with usable AST data did not differ from participants with unusable data in temperament status, age, pubertal status, symptoms of total anxiety or social anxiety per the Screen for Child Anxiety Related Emotional Disorders (Birmaher et al., 1997), or symptoms of depression per the Mood and Feelings Questionnaire (Costello and Angold, 1988) (*p*s>.10). Of 108 participants with usable AST data, 77 also had usable fMRI data. Reasons for missing fMRI data included failure to complete the fMRI task or scan (*n* = 6), unusable fMRI data due to excess motion in the scanner (*n* = 19), falling asleep in the scanner (*n* = 5), and an incidental finding that compromised analyses (*n* = 1). Participants with usable fMRI data did not differ from participants with unusable fMRI data in temperament status, age, pubertal status, or symptoms of total anxiety, social anxiety, or depression (*p*s≥.40).

Thus, a total of 77 participants (49 high in shy/fearful temperament; *M*_age_ = 12.29 years, *SD* = .78 years) were included in the analysis linking attention bias scores to neural activity. These 77 participants did not differ from the rest of the recruited sample (*n* = 52) in temperament status, age, pubertal status, or symptoms of total anxiety, social anxiety, or depression (*p*s>.40). Most included participants identified as White, non-Hispanic (74.0 %); 15.6 % identified as Black or African American (non-Hispanic), 1.3 % identified as Asian, 9.1 % identified as biracial, and 5.2 % reported Hispanic or Latino origin. Income was reported by parents using an 11-point interval scale. Median total family income was between $80,001 and $90,000; 41.6 % of the sample reported annual incomes above $100,000.

This work is part of a larger body of research testing how threat sensitivity in early adolescence confers risk for social anxiety and depression in mid- to late-adolescence. Given that the focus of this manuscript is on concurrent brain-behavior associations, and there is an unbalanced number of high shy/fearful and low-moderate shy/fearful participants in the sample with usable data for this manuscript, we do not report on potential individual differences related to temperament.

### 2.2 Procedure

The study was approved by the University of Pittsburgh Institutional Review Board. The study occurred in three laboratory visits. During Visit 1, parents provided informed consent and youth provided informed assent to participate in the study and the WASI was administered by a research assistant. The K-SADS-PL (parent and child interviews; Kaufman et al., 2016) was then administered to each participant and her primary caregiver separately by trained interviewers (master’s/doctoral level clinicians) to determine current and past DSM-5 diagnoses. During Visit 2, which took place about a month following Visit 1 (mean = 4.88 weeks, SD = 2.89 weeks), participants returned to the laboratory, where they completed the AST and first component of the Chatroom Interact task. Visit 3 occurred several weeks following Visit 2 (mean = 8.12 weeks, SD = 4.58 weeks). During Visit 3, participants completed the fMRI portion of the Chatroom Interact task (see below) in an MRI scanner.

#### 2.2.1 fMRI acquisition

Scanning took place on a 3 T Siemens Prisma magnet with a 64-channel phase array coil. Task stimuli were projected onto a color, high-resolution LCD screen in front of the scanner bed and viewed in a mirror mounted on the head coil. Head movement was constrained by foam padding. Participants were equipped with a response glove on their right hand that allowed them to make responses during the task. All included participants were right-handed. Anatomical images covering the entire brain were acquired first using a three-dimension magnetization-prepared rapid gradient-echo T1-weighted sequence (repetition time [TR] = 2300ms, echo time [TE] = 3.93 ms, flip angle 9°, inversion time [TI] = 900ms, voxel size = 1mm^3^). Functional images were acquired using multi-band gradient echo-planar (EPI) sequences (60 slices, three-factor multiband) sensitive to BOLD contrast [T2*] (TR = 1500ms, TE = 30ms, flip angle 55°, voxel size = 2.3 × 2.3 × 2.3 mm). Field maps were acquired using gradient echo planar imaging sequence for correction of field distortions in the functional images with the following parameters: TR = 590ms, TE1 = 4.92 ms, TE2 = 7.38 ms, voxel size = 2.3 × 2.3 × 2.3 mm, ﬂip angle 60°. Participants then completed the Chatroom Interact task.

### 2.3 Measures

#### 2.3.1 Attention speech task

Participants completed the AST during Visit 2. The AST was designed as a novel measure of attention bias in a real-world, socially evaluative context (Allen et al., 2019). In this task, a research assistant instructed participants to give a two-minute speech. Participants were asked to pretend that they were auditioning for a reality TV show for teens and explain why they should be picked for the show. Participants were given examples of what they could talk about, such as how smart, likeable, or fun they are. Participants were given two minutes beforehand to prepare their speech with their participating parent and were told that two judges would be evaluating their speech.

When it was time for the speech to begin, the parent was seated behind the participant and two judges (both of whom were young adult female study confederates) walked in silently with clipboards and took seats opposite the participant. A bell indicated that participants could begin their speech. The two judges were previously trained to act in predetermined ways, either positive or potentially critical. The positive judge smiled at the participant and took notes at designated intervals. The potentially critical judge maintained a neutral face throughout the speech, took intermittent notes, shuffled her feet, and spent time looking away and toward the participant at designated intervals. Due to ethical considerations, the potentially critical judge did not display overtly negative expressions or behavior; however, research suggests that neutral faces are often interpreted as negative in the context of social evaluation (e.g., Wieser and Brosch, 2012). The locations of the positive and potentially critical judges in the room were counterbalanced across participants.

After the speech, girls were provided with pictures of each judge and asked to complete a questionnaire evaluating how stressed and happy each judge made them feel on a 0–10 Likert scale, with 10 being the most stressed or happy. This questionnaire was included as a manipulation check to ensure that participants were viewing the potentially critical judge more negatively compared to the positive judge. Previous research using this questionnaire with this task has found participants to report significantly more stress and less happiness in response to the potentially critical judge compared to the positive judge (Woody et al., 2019).

##### 2.3.1.1 Eye tracking glasses for attention speech task

Participants wore Tobii Pro Glasses 2 (Tobii Technology, Inc., Falls Church, VA) to track attention throughout the speech task. These wearable eye-tracking glasses are made to look and feel similar to reading glasses, though they are larger and heavier than typical reading glasses. They have four eye tracking sensors with a sampling rate of 50 Hz and infrared illuminators to support the eye tracking sensors. Additionally, the glasses have a high-definition camera (located above the nose) to capture the participant’s visual field, which extends approximately 80° horizontal and 52° vertical, in order to map the location of the participant’s gaze onto what the participant is viewing. Tobii’s standard software was used to estimate the eye’s position and gaze point.

Before beginning the AST, participants completed a calibration procedure. Participants were instructed to look at a specific target on a small card in front of them. A research assistant completed the calibration procedure on a tablet that received information from the glasses. To ensure that the calibration procedure was correct, a research assistant asked participants to look at various objects in the room while checking that the gaze point was accurate on the tablet.

#### 2.3.2 Chatroom interact task

The Chatroom Interact Task, which mimics peer interactions in the form of an online chatroom, was used to model neural responses to peer rejection and acceptance (Silk et al., 2012b, 2014). The first component of the task was completed in the laboratory at Visit 2. At this visit, participants meet virtual “peers,” girls whom they believe to be participants in the study, and choose five girls they would most like to interact with during the fMRI scan. Participants are then matched with two of these girls for the scan and the participant and peers take turns selecting who they would rather talk to about different interests (e.g., music, sports).

The MRI scan took place at Visit 3. The fMRI portion of the Chatroom Interact task consists of four blocks with 15 trials in each block, with a total run time of 15.1 min. Throughout the fMRI task, pictures of participants’ faces are shown two at a time. The first block consists of control trials; in each control trial, a dot appears over one of two faces on the screen and participants are asked to press a button to indicate which side of the screen the dot is on. The second block consists of “participant’s choice” trials; participants choose which girl they would rather chat with about rotating topics. In the third and fourth blocks, the participant is chosen/not chosen by the virtual peers; in one block, participants are “rejected” in 2/3 of trials and in the other participants are “accepted” in 2/3 of trials. During “rejection” trials, a peer selects the other peer to chat with, and a large ‘X’ is superimposed on the participant’s picture. During “acceptance” trials, a peer selects the participant, and the participant’s picture is highlighted. Each trial is 15 s long; the topic is presented for 3 s and feedback for 12 s. The participant assigned to make the choices for each block is also presented for 1.5 s at the start of each block. To maintain task engagement, participants are asked to press a button to indicate which person is chosen during the third and fourth blocks. At the end of the task, participants rated how happy, sad, and excluded they felt when they were chosen and not chosen by their peers.

### 2.4 Data analysis

#### 2.4.1 Preliminary analyses

Internal reliability for the AST was measured by computing within-subjects split–half correlations with Spearman–Brown coefficients for attentional bias scores. Paired samples *t*-tests were used to examine differences in how stressed and happy participants felt when viewing the potentially critical judge relative to the positive judge. Paired samples *t*-tests were also used to examine differences in how happy, sad, and excluded participants felt when they were chosen by their peers relative to when they were not chosen on the Chatroom task.

#### 2.4.2 Eye-tracking analysis

Eye tracking data were processed using Tobii Pro Glasses Analyzer (Tobii Technology, Inc., Falls Church, VA). A custom fixation was used to classify eye movements (e.g., fixations, saccades) based on previous research using this technology (Allen et al., 2019; Woody et al., 2019). Fixations were identified by a consecutive chain of raw data points below the velocity threshold of 30 degrees/second. Tobii’s automated “Real-World Mapping” procedure superimposed fixations onto a still snapshot of the participant's field of view when looking at the judges, which was created using a representative still image from each participant’s glasses camera. To ensure accuracy of this procedure, a trained research assistant manually checked whether the location of each fixation point on the video captured by the glasses’ camera appeared in the same location as the fixation point projected onto the still snapshot and corrected the fixation point if necessary. The checking procedure ensured that fixations that did not match up to the still image because of errors due to movement were corrected for. On average, manual fixes were made for 15.35 % of data points across the participants who were included in the final analysis (further information on checking procedure is provided in supplementary material from Woody et al., 2019 and Allen et al., 2019). An area of interest (AOI) was then created around each judge (the entire head and body) to identify whether the participant’s eye gaze was fixated on either judge at each sampling point on the representative still image. Both the head and body were included in the AOI because judges were instructed to use facial (e.g., smiling) and body language cues (e.g., crossing legs) of approval and disapproval. Attention index was derived by quantifying “visits” to each judge, which are defined as the time interval between the first fixation on the active AOI (i.e., one of the two judges) and the end of the last fixation within the same active AOI where there have been no fixations outside the AOI. Following previous procedures (Woody et al., 2019), participants were excluded from the dataset if they had less than 50 % valid gaze data, as determined by Tobii software. Participants with usable data had an average of 83.54 % valid gaze data (SD = 11.82, range = 50.68–98.27 %). In the current study, we focused on sustained attention capture, which we measured by examining the total duration of visits to each judge across the two-minute speech (i.e., visit time or dwell time), creating a visit time bias score (i.e., total visit duration on potentially critical judge – total visit duration on positive judge), which may be interpreted as difficulty disengaging attention from the potentially critical judge.

#### 2.4.3 fMRI data preprocessing

fMRI data were preprocessed according to standard protocols based on the general linear model, using a canonical hemodynamic response function and Statistical Parametric Mapping software (SPM12; Wellcome Department of Cognitive Neurology, London, UK). The preprocessing procedure includes 1) Image reconstruction and reorientation to the anterior and posterior commissure line, 2) Generation of motion parameter files and distortion correction using a voxel displacement map, 3) Co-registration with the high-resolution structural image, 4) Segmentation of the anatomical images into gray and white matter maps, 5) Spatial realignment and normalization to a standard Montreal Neurological Institute (MNI) T1 template with 2 mm voxels, 6) Spatial smoothing using a 6 mm full-width at half-maximum Gaussian kernel, and 7) Use of ArtRepair (Mazaika et al., 2009) to detect head motion artifact and make appropriate adjustments. Scans with > 0.5 mm of incremental motion, > 3 mm from the baseline image, and/or 3 standard deviations [SD] intensity shifts were considered outliers. Outlier scans were replaced with a linear interpolation between the two nearest non-outlier scans. Any subjects with more than 25 % of volumes with excess movement were excluded (i.e., censored) from analyses.

#### 2.4.4 Functional connectivity analysis

The CONN toolbox for SPM (Whitfield-Gabrieli and Nieto-Castanon, 2012) was used to run seed-to-voxel connectivity analyses. Psychological regressors included effects of task (feedback anticipation, acceptance feedback, rejection feedback, control, participant’s choice). Six head realignment motion parameters were included as nuisance regressors for each participant, and physiological noise from white matter and cerebrospinal fluid was also regressed out for each participant (Behzadi et al., 2007). Linear de-trending was applied for additional denoising and a .008–.09 Hz temporal band-pass filter was applied to minimize effects of low-frequency drift and high-frequency noise.

We used CONN’s default for functional connectivity analyses, a first-level weighted generalized linear model (GLM) for weighted correlation measures of condition-specific associations between the amygdala seed BOLD timeseries and each voxel in the prefrontal cortex ROI mask. Each condition of interest was modeled with a boxcar function and convolved with the canonical hemodynamic response function; this defined condition-specific weights. This ROI mask was created using the Talairach Demon frontal lobe ROI mask (53779 voxels) created using the WFU PickAtlas Tool (v3.0.5b). While ventromedial PFC regions play a strong role in emotion regulation (Etkin et al., 2011), more lateral PFC regions are frequently implicated in attentional processes (Pine and Fox, 2015). Thus, we included a more encompassing PFC mask.

While weighted GLM analysis can provide “relative” measures of functional connectivity, comparing connectivity in one condition relative to another, this analysis also provides “absolute” measures of functional connectivity during a single task condition, using a nonparametric estimation of weighted correlation measures within each single condition (e.g., Poletti et al., 2018; Belleau et al., 2020). This approach (“absolute” connectivity) tests the hypothesis that connectivity within a single task condition is consistent among the sample and differs significantly from zero (tested using a one-sample *t*-test; Poletti et al., 2018). Examining functional connectivity within a single condition may be particularly useful in individual difference research, given research suggesting that neural difference scores (i.e., neural activation in one condition relative to another) shows poorer reliability than neural activation within a single condition (Infantolino et al., 2018). To generate amygdala-PFC correlation maps during rejection feedback, the time series was extracted separately from right amygdala seed (2709 mm^3^) and left amygdala seed (2606 mm^3^), defined anatomically using the default atlas in the CONN toolbox (derived from the FSL Harvard-Oxford maximum likelihood subcortical atlas; Whitfield-Gabrieli & Nieto-Castañón, 2012), and correlated with every other voxel in the frontal lobe. The seed to frontal lobe correlation maps were normalized using a Fischer’s *z* transformation and used in group-level statistics.

A second-level regression analysis was then used to examine associations between in vivo attention bias scores and amygdala-seeded connectivity during rejection feedback. This analysis was run separately for the left and right amygdala. Hypothesis tests were corrected for multiple comparisons using small volume correction within this mask at a *p* < .005 voxel-wise threshold and applying the false discovery rate to resulting clusters. Analyses were run with and without age as a covariate. Sensitivity analyses were run to test specificity to rejection feedback (see online supplement). Briefly, we examined associations between attention bias scores and amygdala-seeded functional connectivity for the rejection feedback > acceptance feedback contrast (a measure of “relative” functional connectivity) and during acceptance feedback alone.

Supplemental whole-brain analyses testing associations between attention biases and amygdala-seeded connectivity, as well as analyses testing associations between attention biases and basic functional neural activation across the whole brain for the rejection feedback > acceptance feedback contrast, can be found in the online supplement. Exploratory correlational analyses linking attention bias scores, fronto-amygdala connectivity, and self-reported anxiety symptoms can also be found in the online supplement (Table S1).

## 3 Results

### 3.1 Preliminary behavioral findings

#### 3.1.1 AST

Spearman-Brown coefficients were found to be acceptable for all bias scores and raw score indices (all coefficients above 0.78; Table 1). On average, adolescent girls spent more time fixating on the positive judge, relative to the potentially critical judge, during the speech task, with a mean attention bias score of -5.64, standard deviation of 17.63, and range from -74.41–44.87, suggesting meaningful variability.

**Table 1:** Split-half reliability using Spearman Brown coefficients for attention indices.

	Mean (SD)	Spearman Brown Coefficient for block 1 and 2
**Speech Task Indices (s)**		
Total Visit Time Bias Score	−5.64 (17.63)	0.86
Total Visit Time on Positive Judge	13.29 (16.35)	0.93
Total Visit Time on Potentially Critical Judge	7.65 (9.35)	0.79

*Note.* Spearman Brown coefficient was calculated using the first half compared to the second half of the speech (approximately 61 s each). Split-half reliability <.60 is considered to be unacceptable.

Paired samples *t*-tests revealed that on average, participants reported (immediately following the AST) that the potentially critical judge made them feel more stressed (*t*(105) = 4.29, *p* < .001, Cohen’s *d* = .42) and less happy (*t*(105)=-6.07, *p* < .001, Cohen’s *d* = .59) than the positive judge. Attention bias scores were not significantly associated with age (*r*=-.11, *p* = .26).

#### 3.1.2 Chatroom interact task

Paired samples *t*-tests revealed that on average, participants felt less happy (*t*(74)=-8.69, *p* < .001, Cohen’s *d* = 1.00) and more excluded (*t*(74) = 2.52, *p* = .014, Cohen’s *d* = .29) when they were not chosen by their peers relative to when they were chosen. On average, participants did not differ in level of sadness when they were not chosen, relative to when they were chosen (*t*(74) = .20, *p* = .844, Cohen’s *d* = .02).

### 3.2 Associations between in vivo attention biases towards social threat and amygdala-PFC functional connectivity to social rejection

**Fig. 1:**
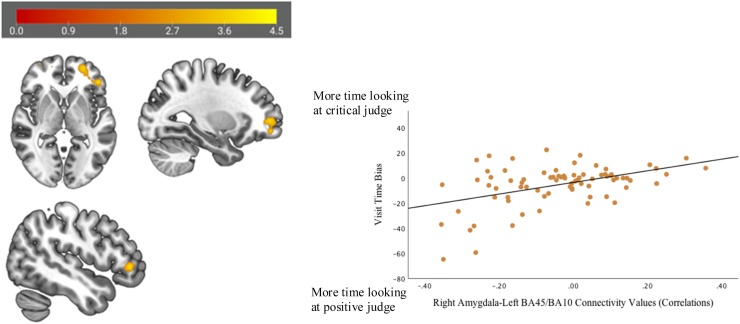
Attention bias towards potential social rejection during an in vivo speech task correlated significantly with functional connectivity between the right amygdala (anatomically defined) and left BA45/BA10 (pictured below; cluster size = 2624 mm^3^) during social rejection feedback on the Chatroom Interact task. The correlation is displayed for reference.

**Fig. 2:**
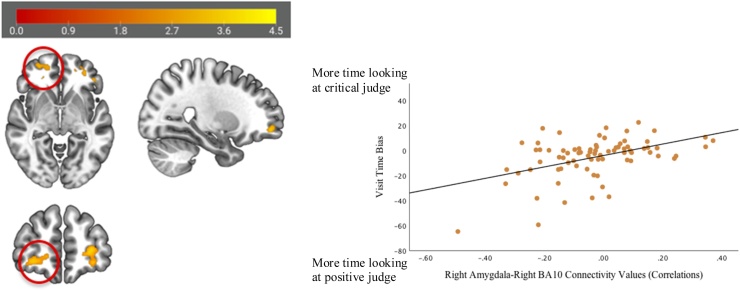
Attention bias towards potential social rejection during an in vivo speech task correlated significantly with functional connectivity between the right amygdala (anatomically defined) and right BA10 (pictured below; cluster size = 1648 mm^3^) during social rejection feedback on the Chatroom Interact task. The correlation is displayed for reference.

Significant positive correlations between attention bias scores and functional connectivity during rejection feedback were found between the right amygdala (anatomically defined) and two clusters in the bilateral PFC: 1) left BA45/BA10 (cluster size = 2624 mm^3^; peak x,y,z=-48,38,0 (additional peaks include -26,56,0 and -40,36,2); *t*(75) = 4.04, *p*_FDR_ = .004; Fig. 1) and 2) right BA10 (cluster size = 1648 mm^3^; peak x,y,z = 18,50,-10 (additional peaks include 28,54,-8 and 20,42,-8); *t*(75) = 4.34, *p*_FDR_ = .019; Fig. 2). A positive correlation suggests that adolescent girls attending more to the potentially critical judge relative to the positive judge during an in vivo social stress task also showed more positive (or less negative) coupling between the right amygdala and PFC; girls attending more to the positive judge relative to the potentially critical judge showed greater negative coupling between the right amygdala and PFC.

Findings held controlling for age (left BA10: 2768 mm^3^; right BA10: 1360 mm^3^). Additionally, right amygdala-PFC connectivity values resulting from the primary analysis were not significantly correlated with age (*ps*>.05; Table S1, online supplement). No significant findings emerged for the left amygdala seed and no additional findings emerged in supplemental whole-brain analyses.

Sensitivity analyses revealed that the positive association between attention bias scores and right amygdala-left BA10 functional connectivity replicated for the rejection > acceptance contrast but did not surpass cluster-level FDR correction (left BA10 cluster size = 872 mm^3^; peak x,y,z=-28,54,2; *t*(75) = 4.65, *p*_FDR_ = .197). In addition, a positive association between attention bias scores and functional connectivity between the right amygdala and right BA10 during acceptance feedback alone was found (right BA10 cluster size = 3072 mm^3^; peak x,y,z = 20,64,-2; *t*(75) = 4.65, *p*_FDR_ = .001; Figure S1, online supplement).

## 4 Discussion

The present study uses multiple novel methods to examine potential “real-world” implications of less negative fronto-amygdala connectivity while adolescent girls oversampled for shy/fearful temperament (a risk factor for social anxiety) receive social evaluative feedback from peers. Importantly, reliability for a new in vivo attention task was supported, a key contribution of the present study. Findings support the ease of implementation and reliability of this ecologically valid attention task in the present population. These results are especially important given concerns about the reliability of the traditional dot-probe task (e.g., Price et al., 2015, 2019). This is the first large-scale investigation of the AST that demonstrates adequate reliability of the AST, supporting its use in future research.

Associations between real-world attention biases and patterns of fronto-amygdala connectivity on the Chatroom task may provide insight into neural mechanisms supporting sensitivity to social and affective stimuli in adolescence (e.g., Crone and Dahl, 2012; Somerville, 2013). Aligning with hypotheses, girls who attended more to a potentially critical judge relative to a positive judge during the AST also showed more positive coupling between the right amygdala and bilateral anterior PFC while receiving social rejection feedback on the Chatroom task. Interestingly, similar prior research has implicated the right amygdala specifically (e.g., Gee et al., 2013; Price et al., 2016; Abend et al., 2020), which could suggest meaningful lateralization. However, lateralized effects could also be related to inadvertent effects from data analysis (Murphy et al., 2020).

The regions of prefrontal cortex found to correlate with amygdala activity during rejection feedback were in more lateral portions of the anterior PFC (BA 10) and inferior frontal gyrus. The specialized, dissociable functions of medial and lateral sub-regions of BA 10 have been a topic of research interest over the past couple decades (e.g., Burgess et al., 2007; Gilbert et al., 2006a, b; Peng et al., 2018). Clusters identified in the present study are located in spatially similar locations as previous PFC regions implicated in executive functioning and attention (Van Overwalle, 2009; Gilbert et al., 2007), as well as in emotion control (Kaldewaij et al., 2021) and emotion regulation (Morawetz et al., 2017). Functional connectivity between the amygdala and lateral PFC has also been implicated in a variety of attention tasks (LeDoux and Pine, 2016). The lateral PFC may engage attention-regulatory functions to maintain goal-directed actions and compensate for heightened amygdala reactivity to threat (Pine & Fox, 2015). Additionally, structural connections between the lateral PFC, amygdala, and vmPFC may provide a pathway through which emotion influences attentional systems (Vuilleumier, 2005), and/or the lateral PFC may modulate amygdala activity through connections with ventromedial prefrontal regions (Etkin et al., 2011; Urry et al., 2006). Thus, in the present study, less negative (and more positive) amygdala-PFC functional connectivity in girls with higher attention biases towards social evaluative threat in the real world may represent reduced prefrontal down-regulation of the amygdala’s response to negative social evaluation.

Unexpectedly, however, findings were not specific to social rejection feedback; sensitivity analyses revealed that girls with greater attentional biases towards potential threat in the real world also showed more positive fronto-amygdala coupling during social acceptance feedback in the scanner. The amygdala responds to both appetitive and aversive stimuli that are emotionally arousing (e.g., Murray, 2007; Shabel and Janek, 2009) and may influence spatial attention to stimuli signaling threat and reward (Peck and Salzman, 2014). Interactions between the amygdala and anterior PFC may thus work to regulate attention in the presence of emotionally salient stimuli, regardless of valence. Further, in the present study (using a non-clinical sample of adolescents), acceptance and rejection feedback from peers on the Chatroom task could be comparable in emotional salience, eliciting similar patterns of fronto-amygdala connectivity. Present findings may suggest that girls with more positive amygdala-PFC coupling to salient social feedback in the MRI scanner struggle to regulate their emotional and attentional responses to salient, emotionally arousing social feedback during the social speech task (i.e., spend more time attending to the potentially critical judge than the positive judge).

Despite the narrow age range of the present study, these findings could have implications for understanding developmental shifts in fronto-amygdala connectivity believed to occur during adolescence. Silvers et al. (2017) suggest that lateral PFC-amygdala connectivity involved in regulating responses to negative social cues may be slower to develop in adolescence than other neural systems involved in emotion regulation, including vmPFC-amygdala connectivity. More positive coupling between the amygdala and PFC in the present study could thus represent a more immature pattern of connectivity signaling reduced prefrontal regulation and contributing to heightened sensitivity to negative social evaluation in early adolescence. As this sample was recruited for an ongoing longitudinal study, future research in this sample will examine developmental trajectories of fronto-amygdala connectivity and biased attention to potential social evaluative threat. One possibility that can be tested in future research is that more immature patterns of fronto-amygdala coupling supporting attention biases towards social threat confers risk for future anxiety in youth at higher risk. This could be supported by research showing differential patterns of threat-related amygdala-dlPFC connectivity in young adults who differed in behaviorally inhibited temperament in childhood (Hardee et al., 2013).

The present study is strengthened by the unique sample composition. Early adolescent girls are an important population in which to study associations between attention bias to social threat and brain function. Adolescent girls show hypersensitivity to social evaluation (Rudolph and Conley, 2005) and are at high risk for social anxiety (Merikangas et al., 2010), and attention biases towards threat may be one mechanism contributing to this risk (Pintzinger et al., 2017). However, it remains unknown whether findings might extend to adolescent boys. Future research is needed to examine similar processes in at-risk boys and more diverse samples. Additionally, the narrow age range of the sample (11–13 years) may prohibit our present ability to speak to age-related effects at present; however, the longitudinal design of the study will enable these analyses in the future. Future research is needed to more clearly delineate the specific contributions of PFC and amygdala sub-regions to processing social feedback in adolescence.

Task-related limitations are also worth noting. First, while the AST shows clear improvements in ecological validity over the standard computerized dot-probe task, the speech task still occurs in the laboratory, an inherent confound that could influence participants’ behaviors. Second, participants’ primary caregivers were in the room during the speech; although the caregivers were located behind the participants and instructed not to speak during the task, their presence may be another confound. Additionally, the Chatroom task is an interactive online platform that simulates basic social media interactions, supporting its ecological validity and salience for the adolescent population. However, the task might not capture some nuances of social rejection for today’s youth (e.g., not receiving a “like” on a picture). Future research continuing to test and improve the ecological relevance of these paradigms is important.

## 5 Conclusion

In the present study, adolescent girls who attended more to a potentially critical judge relative to a positive judge during a novel, in vivo attention bias task showed more positive functional connectivity between the right amygdala and anterior PFC during social evaluative feedback on the Chatroom task, potentially signaling poorer prefrontal regulation. Findings provide real-world correlates of fMRI findings and potential insight into the neural circuitry supporting modulation of attention in the context of salient social-emotional stimuli in adolescent girls. Findings also support the continued use of this in vivo attention bias task in developmental research. Given the narrow age range of the sample and cross-sectional nature of the study, longitudinal research is needed to test how attention biases and corresponding patterns of amygdala-anterior PFC connectivity might change throughout development and confer risk for future psychopathology.

## Data statement

Behavioral and neural data from this study are not currently publicly available but are available for study participants who consented to the public use of their data upon request. Please contact the corresponding author for more details.

## Funding

This project was supported by 10.13039/100000025National Institute of Mental Health grant R01 MH103241(MPIs: J.S. Silk and C.D. Ladouceur) and a National Science Foundation Graduate Research Fellowship awarded to S.L. Sequeira under Grant No. 1747452.

## Declaration of Competing Interest

The authors report no declarations of interest.
