# Supplementary material for: “Don’t judge me!”: Links between in vivo attention bias toward a potentially critical judge and fronto-amygdala functional connectivity during rejection in adolescent girls
Source: Dev Cogn Neurosci. 2021 May 5;49:100960. doi: 10.1016/j.dcn.2021.100960 (PMC8120940; doi:10.1016/j.dcn.2021.100960)
Supplement: Supplementary file 1 [file mmc1.docx]

Online Supplement

**SUPPLEMENTAL FUNCTIONAL CONNECTIVITY ANALYSES**

Two additional first-level connectivity analyses were conducted in CONN to test whether findings were specific to neural activity during rejection. First, the same approach taken to examine amygdala-seeded connectivity during rejection feedback (i.e., “absolute”
 measures of connectivity) was used to examine amygdala-seeded connectivity during acceptance feedback. Second, a more traditional approach was taken to examine how attention biases are associated with differences in amygdala-PFC connectivity to rejection feedback relative to acceptance feedback (i.e., rejection > acceptance contrast) and acceptance feedback relative to rejection feedback (i.e., acceptance > rejection contrast). A first-level weighted generalized linear model (GLM) was used for the purpose of comparing amygdala-seeded connectivity between conditions.

**SUPPLEMENTAL WHOLE-BRAIN NEURAL ACTIVATION ANALYSES**

Secondary whole-brain analyses were conducted in the CONN toolbox to explore additional associations between attention biases and amygdala-seeded connectivity during rejection feedback and acceptance feedback. No additional findings emerged from this analysis. The findings resulting from the primary ROI analysis examining regions of the PFC that correlate with amygdala activity during rejection feedback (i.e., left BA45/BA10, right BA10) survived cluster-level correction in whole-brain analyses (cluster-level *p*_FDR_=.004 for left BA45/BA10, *p*_FDR_=.048 for right BA10), as did the findings resulting from the primary analysis examining amygdala-PFC connectivity during acceptance feedback (i.e., right BA10, *p*_FDR_=.004).

Second-level regression analyses were implemented using SPM12 to examine associations between attention bias scores and basic neural activation across the whole brain for the rejection feedback > acceptance feedback contrast, with a voxel-wise threshold of *p*<.005 and cluster-level threshold of *p*_FDR_<.05. No significant associations between attention bias scores and basic neural activation (across the whole brain) emerged for the rejection>acceptance contrast.

**ASSOCIATIONS BETWEEN FRONTO-AMYGDALA CONNECTIVITY, ATTENTION BIASES, AND SELF-REPORTED ANXIETY SYMPTOMS**

Correlations between self-reported anxiety symptoms, attention biases, and fronto-amygdala connectivity values resulting from the primary analysis were explored given prior research linking attention biases and fronto-amygdala connectivity to anxiety in adolescence (e.g., White et al., 2017. A 44-item SCARED (Birmaher et al., 1997)-Child version was completed by adolescents at Visit 1 to assess anxiety symptoms. The SCARED assesses symptoms of social anxiety, generalized anxiety, separation anxiety, school avoidance, and panic disorder, which can be combined into a total anxiety score (Cronbach’s $\alpha$=.91). Each item is rated on a 3-point Likert-type scale. This version of the SCARED included three additional items to assess social anxiety, resulting in a maximum total score of 88 and social anxiety score of 20. Total anxiety scores and social anxiety subscale scores were used in exploratory analyses.

As depicted in Table S1, total anxiety severity from the SCARED was not significantly associated with attention biases or right amygdala-PFC connectivity during rejection or acceptance feedback (*ps*>.20). Social anxiety was also not significantly associated with attention biases or amygdala-PFC connectivity values during rejection feedback (*p*s>.20). A small but nonsignificant negative correlation was seen between social anxiety scores and amygdala-PFC connectivity during acceptance feedback (*r*=-.19, *p*=.093).

**Table S1**

***Correlation Table (N=77)***

|  | 1 | 2 | 3 | 4 | 5 | 6 | 7 |
| --- | --- | --- | --- | --- | --- | --- | --- |
| 1. Age | 1 |  |  |  |  |  |  |
| 2. Total anxiety | .10 | 1 |  |  |  |  |  |
| 3. Social anxiety | -.04 | .82** | 1 |  |  |  |  |
| 4. Attention bias | -.12 | -.01 | -.13 | 1 |  |  |  |
| 5. Right amygdala-left PFC  connectivity (rejection) | -.01 | .00 | .01 | .46** | 1 |  |  |
| 6. Right amygdala-right PFC  connectivity (rejection) | -.20^y^ | .02 | .06 | .46** | .67** | 1 |  |
| 7. Right amygdala-right PFC  connectivity (acceptance) | -.22^y^ | -.13 | -.19^y^ | .52** | .30* | .44** | 1 |
| Mean | 12.29 | 16.68 | 3.12 | -5.46 | -.04 | -.03 | -.02 |
| Standard Deviation | .78 | 10.99 | 2.36 | 15.95 | .16 | .16 | .14 |
| Range | 11-13 | 0-44 | 0-11 | -64-22 | -.36-.36 | -.49-.37 | -.46-.23 |

*Note.* ^y^*p*<.10, **p*<.05, ***p*<.001; Anxiety symptoms were assessed using the SCARED – Child report. Attention bias was calculated as the total visit duration on potentially critical judge – total visit duration on positive judge on the AST. Connectivity values are the correlation values resulting from the primary analyses regressing attention bias scores on amygdala-PFC connectivity during rejection feedback or acceptance feedback.

**Figure S1**

***Attention bias towards potential social rejection during an in vivo speech task correlated significantly with functional connectivity between the right amygdala (anatomically defined) and right BA10 (pictured below; cluster size = 3072 mm^3^) during social acceptance feedback on the Chatroom Interact task. The correlation is displayed for reference.***

***
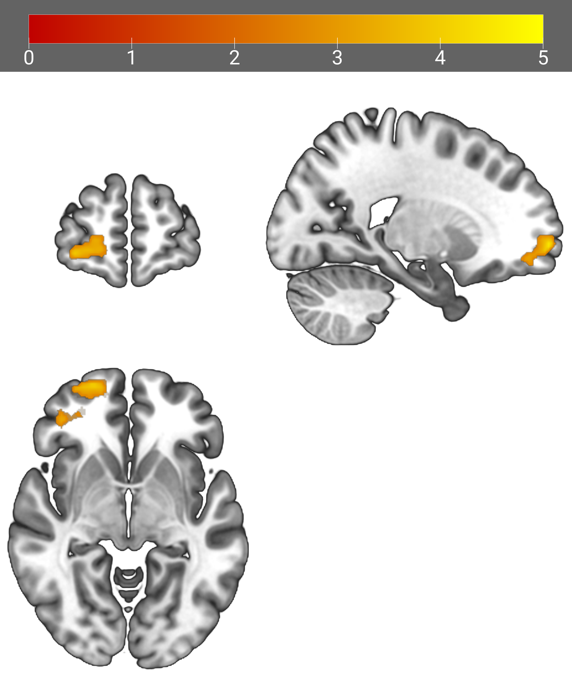
***

***
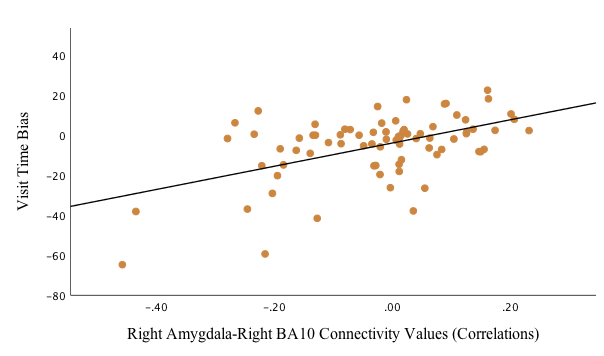
***

More time looking at critical judge

More time looking at positive judge

References

Birmaher, B., Khetarpal, S., Brent, D., Cully, M., Balach, L., Kaufman, J., & Neer, S. M. (1997). The screen for child anxiety related emotional disorders (SCARED): Scale construction and psychometric characteristics. *Journal of the American Academy of Child & Adolescent Psychiatry*, *36*(4), 545-553.

White, L. K., Sequeira, S., Britton, J. C., Brotman, M. A., Gold, A. L., Berman, E., … & Leibenluft, E. (2017). Complementary features of attention bias modification therapy and cognitive-behavioral therapy in pediatric anxiety disorders. *American Journal of Psychiatry*, *174*(8), 775-784.
